# The Complete Mitochondrial Genome of *Petalocephala arcuata* Cai Et Kuoh, 1992 (Hemiptera: Cicadellidae: Ledrinae: Petalocephalini) and Its Phylogenetic Implications

**DOI:** 10.3390/genes16050567

**Published:** 2025-05-10

**Authors:** Yujian Li, Yihong Guo, Ran Li, Yongcheng Liu, Chao Xue, Lina Jiang, Sai Jiang, Wei Wang, Xianfeng Yi

**Affiliations:** School of Life Sciences, Qufu Normal University, Qufu 273165, China; yujian528@163.com (Y.L.); guoyihong719@163.com (Y.G.); li471329014@163.com (R.L.); 17864731918@163.com (Y.L.); xuebhc1001@163.com (C.X.); firstna@163.com (L.J.); js19961120@163.com (S.J.); echo323846@163.com (W.W.)

**Keywords:** mitochondrial genome, *Petalocephala arcuata*, phylogenetic analysis, Cicadellidae

## Abstract

Background/Aims: Ledrinae comprises about 460 described species across five tribes and represents an early-branching, morphologically distinctive lineage of leafhoppers, yet its intra-subfamilial relationships remain ambiguous owing to limited mitogenomic sampling. Here, we sequence and annotate the complete mitochondrial genome of *Petalocephala arcuata*—only the 18th Ledrinae mitogenome—to broaden taxon coverage within the genus and furnish critical molecular data for rigorously testing Ledrinae monophyly and refining tribal and genus level phylogenetic hypotheses. Methods: In this study, we sequenced and annotated the complete mitochondrial genome of *P. arcuata* via Illumina sequencing and de novo assembly, and reconstructed the phylogeny of 62 Cicadellidae species using maximum likelihood and Bayesian inference methods. Results: The 14,491 bp circular mitogenome of *P. arcuata* contains 37 genes with 77.4% A+T. All PCGs use ATN start codons except *ND5* (TTG), and codon usage is A or U biased. Of 22 tRNAs, only *trnS1* lacks a DHU arm, while the others adopt the canonical cloverleaf structure. Bayesian inference and maximum likelihood analyses produced broadly congruent topologies with mostly high nodal support, recovering Ledrinae as monophyletic and clustering all *Petalocephala* species into a well-supported clade. Conclusions: In this study, we enriched the molecular resources for the genus *Petalocephala* by sequencing, annotating, and analyzing the complete mitochondrial genome of *P. arcuata*. Phylogenetic reconstructions based on these genomic data align closely with previous morphological diagnoses, further confirming the monophyly of the genus *Petalocephala*.

## 1. Introduction

The subfamily Ledrinae (Hemiptera: Auchenorrhyncha: Cicadellidae) comprises five tribes—Ledrini, Rubrini, Xerophloeini, Afrorubrini, and Hespenedrini—and currently encompasses approximately 460 described species. Members of this lineage occur worldwide, with particularly high diversity in the Oriental region and Australia [1,2,3,4,5]. Li et al. [6] have identified China as a principal center of Ledrinae diversity, recording 28 genera and over 180 species within its borders. Owing to its early-branching position within Cicadellidae and its suite of distinctive morphological characters, Ledrinae has long been a focal group for taxonomic and phylogenetic investigations [7,8]. To date, most studies have been confined to α-taxonomy, relying on morphological diagnoses to describe new taxa; however, pronounced intraspecific variation in certain species has frequently led to taxonomic ambiguities and misidentifications [9,10].

Compared with traditional morphology-based taxonomy, molecular phylogenetic analyses effectively surmount the subjectivity inherent in morphological character scoring and the challenges of homology assessment. Single locus markers (e.g., 28*S rDNA* [11], *COI* [12]) often yield biased topologies due to limited phylogenetic signal and heterogeneous substitution rates. With the advent of high-throughput sequencing [13], a combined strategy—integrating complete mitochondrial genomes with multiple nuclear and mitochondrial loci—has become increasingly favored for its ability to substantially improve tree resolution and nodal support [14,15,16,17,18,19,20]. Mitochondrial genomes offer distinct advantages over nuclear markers: strict maternal inheritance, negligible recombination, and a compact gene arrangement (absence of introns and only one extensive intergenic spacer in the control region) [21]. Furthermore, genome-level characters—such as gene-order rearrangements, intergenic spacer length variation, and unconventional tRNA anticodon usage—provide a wealth of fine-scale phylogenetic signals for disentangling complex lineage relationships [22]. In earlier work, Wang et al. [10] employed mitochondrial, nuclear, and combined datasets to perform a multilocus phylogenetic reconstruction of Ledrinae, with particular emphasis on the internal relationships of its largest tribe, Ledrini. They observed that *Destinoides* and *Petalocephala* are morphologically congruent, prompting Jones and Deitz [2] to synonymize them; however, Sun et al. [23] challenged this synonymy, noting that these types specimens had not been examined. More analyses by Wang et al. [10] revealed that both *Destinoides* and *Petalocephala* are polyphyletic, with *Petalocephala* resolved into two distinct clades. One clade is sister to the *Tituria* + *Laticorona* lineage and can be diagnosed by the elongate apical process of the male genitalia; this lineage was therefore erected as the new genus *Lataramus* (comprising *Lataramus gongshanensis* and *Lataramus dicondylicus*), whose members are readily distinguished by their pronounced genital apical projections. Moreover, phylogenetic analyses of *Petalocephala dicondylica*, *L. gongshanensis*, and *Petalocephala eurglobata*—despite a limited taxon sampling of six Ledrinae genera—yielded a topology consistent with subsequent 2024 studies [10,24], uniformly supporting a non-sister relationship between *L. gongshanensis* and *P. eurglobata*. These congruent results further validate the stability and accuracy of the current subfamilial classification framework.

To date, only 17 complete mitochondrial genomes of Ledrinae have been deposited in the National Center for Biotechnology Information (NCBI) database, representing a marked underrepresentation relative to the number of described taxa. Consequently, expanded taxon sampling within the subfamily is essential to rigorously test its monophyly and to resolve the taxonomic status and interrelationships at the tribal, generic, and species levels. In this study, we sequenced, annotated, and analyzed the complete mitochondrial genome of *P. arcuate* [25], thereby enriching the genomic resources for the genus *Petalocephala*. By integrating these new data with annotations from 16 other validated Ledrinae mitochondrial genomes, we reconstructed a comprehensive phylogenetic framework for the subfamily. Our results provide critical molecular evidence and theoretical support for future phylogenetic and evolutionary studies of Ledrinae and broader Hemipteran lineages.

## 2. Materials and Methods

### 2.1. Sample Collection and DNA Extraction

In August 2022, adult specimens of *P. arcuata* were collected from Malipo County, Yunnan Province, China (22°57′ N, 104°47′ E) (Table 1). All specimens were obtained from natural habitats without the need for collection permits. Upon capture, individuals were immediately preserved in 100% ethanol and subsequently stored at −80 °C in the freezer of the School of Life Sciences, Qufu Normal University. Species identification was performed according to the morphological criteria delineated by Dietrich [26]. To reduce potential contamination from gut microbiota during sequencing library preparation, abdomens were removed and only the remaining abdominal muscle tissue was used for genomic DNA extraction. Genomic DNA was isolated using the SanPrep DNA Gel Extraction Kit (Sangon Biotech, Shanghai, China). Voucher specimens of male genitalia and corresponding DNA samples are deposited in the collections of the School of Life Sciences, Qufu Normal University.

### 2.2. Mitogenome Sequencing, Assembly, Annotation, and Analyses

Genomic DNA extracted from each specimen was subjected to library preparation at Personalbio Inc. (Shanghai, China) and sequenced on an Illumina platform. Raw reads were trimmed of adapters and quality-filtered to yield approximately 2.5 Gb of high-quality sequence data, which were assessed in DNASTAR SeqMan v17.2 and subsequently de novo assembled in Geneious R9.0 [27,28]. The resulting mitochondrial genome assemblies were initially annotated via the MITOS v2.1.9 web server using the invertebrate mitochondrial code; annotations of protein-coding genes (PCGs) and ribosomal RNA genes were further validated by BLAST v2.12.0 comparisons against NCBI, and tRNA genes were confirmed through homology searches against published mitochondrial genomes. Intergenic regions and gene overlaps were manually inspected and delineated, while the secondary structures of 22 tRNA genes were predicted using tRNAscan-SE v1.21 and ARWEN v1.2 [29,30]. Annotated genomes were visualized with Proksee (https://proksee.ca/, accessed on 5 May 2025). For comparative genomic analyses, mitochondrial genome sequences from 59 Cicadellidae species and two Cercopoidea species (as outgroups)—a total of 62 taxa—were retrieved from GenBank and systematically evaluated for nucleotide composition and skew, PCG codon usage and relative synonymous codon usage, and overall genome architecture.

### 2.3. Molecular Phylogenetic Analysis

In this study, 62 mitochondrial genomes from Cicadellidae and the outgroup superfamily Cercopoidea—specifically *C. dorsimacula* and *C. bispecularis*—were analyzed, including the newly sequenced *P. arcuata* (GenBank accession numbers are provided in Table 1). After excising stop codons, the nucleotide sequences of 13 protein-coding genes (PCGs) and two rRNA genes were concatenated for phylogenetic reconstruction. Alignments were generated in MAFFT v7.205: each PCG was aligned codon-by-codon with the L-INS-i algorithm, whereas the two rRNAs were aligned with Q-INS-i to account for secondary-structure constraints [31]. Nucleotide saturation was assessed with DAMBE 5 and found to be non-significant; poorly aligned regions were removed using Gblocks v0.91b with default settings [32]. The filtered alignments were concatenated in PhyloSuite v1.2.3. [33]. Partition schemes and the best-fitting substitution models were selected with ModelFinder [34]. Maximum-likelihood (ML) analysis was performed in IQ-TREE v1.6.12 [35], with branch support evaluated by 5000 ultrafast bootstrap replicates. Bayesian inference (BI) was carried out in MrBayes v3.2.6 [36], employing one cold and three heated chains in two independent MCMC runs of 2,000,000 generations each, sampling every 1000 generations and discarding the first 25% as burn-in. Convergence was accepted when the average standard deviation of split frequencies fell below 0.01 and all key parameters had effective sample sizes (ESSs) greater than 200. The final phylogenetic tree was visualized and taxonomically annotated in iTOL v6.7.4. [37].

## 3. Results and Discussion

### 3.1. Mitogenome Organization and Nucleotide Composition

**Figure 1:**
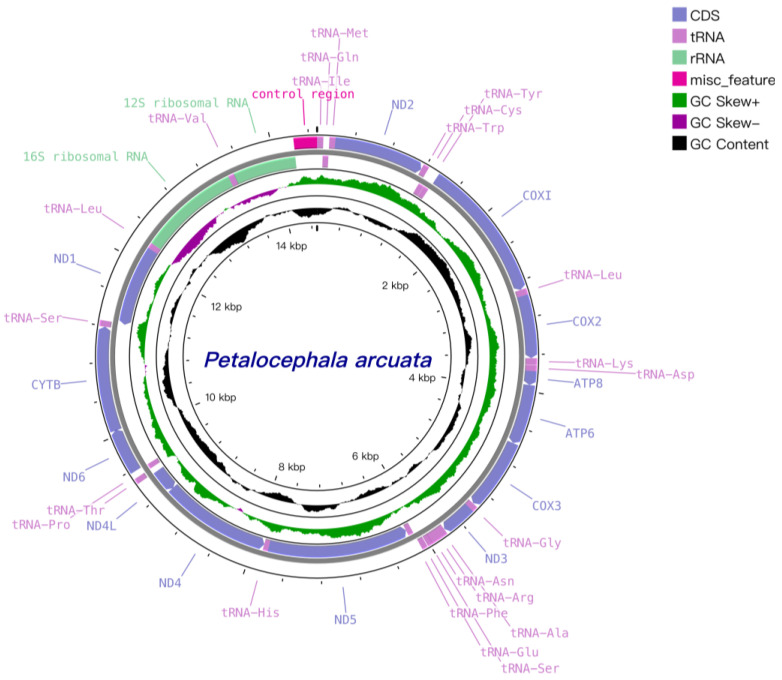
Mitogenomes of *P. arcuata*.

As illustrated in Figure 1 and Table 2, the mitochondrial genome of *P. arcuata* is a 14,491 bp double-stranded circular DNA molecule that harbors the canonical set of 37 mitochondrial genes: 13 protein-coding genes (PCGs), 22 tRNA genes, two rRNA genes and one single A+T-rich control region (CR). Twenty-three genes reside on the heavy (H) strand and fourteen on the light (L) strand. The CR is positioned between *rrnS* and *trnI* and spans 1149 bp (positions 13,342–14,490); variation in its length largely accounts for the differences in mitogenome size observed among Ledrinae species, whose gene order is otherwise conserved.

Nine gene overlaps, totaling 43 bp, were detected, the longest (10 bp) occurring between *trnS2* and *ND1*. Ten intergenic spacers, together comprising 37 bp, were also identified, the longest (8 bp) situated between *trnP* and *ND6*; the lengths of both overlaps and spacers fall within the typical range for congeneric mitogenomes. Notably, the customary 7–8 bp overlap between *Atp8* and *Atp6* reported in other subfamilies is absent in *P. arcuata* and its two congeners [24]. The nucleotide composition is strongly A+T biased, with an overall A+T content of 77.4% and 76.3% in the PCGs. Genome-wide AT-skew and GC-skew values are −0.264 and 0.136, respectively, indicating a relative deficit of adenine versus thymine and an excess of guanine over cytosine (Table 3).

### 3.2. Protein-Coding Genes

The 13 protein-coding genes (PCGs) of *P. arcuata* span 10,923 bp, representing 76.3% of the mitochondrial genome, with an A+T content comparable to that of the full genome. All but one PCG initiate translation with the canonical ATN start codons (ATA, ATG, ATT); only *ND5* employs the non-standard TTG initiation codon. This contrasts with the two congeners analyzed by Wang et al. [24], which uniformly use ATN start sites, suggesting an independent shift in *ND5* initiation within the genus. Termination codons are either complete (TAA or TAG) or truncated (T–), the latter being presumably restored by post-transcriptional polyadenylation.

**Figure 2:**
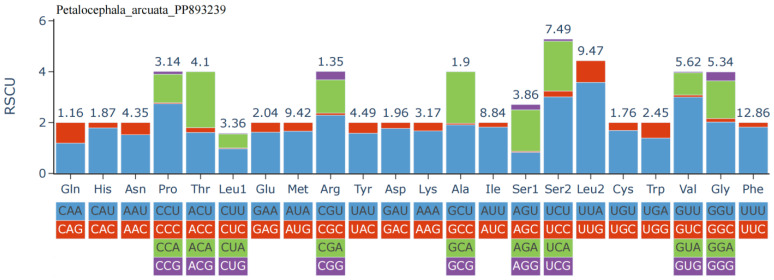
Relative synonymous codon usage (RSCU) of the mitogenome of *P. arcuata*.

**Figure 3:**
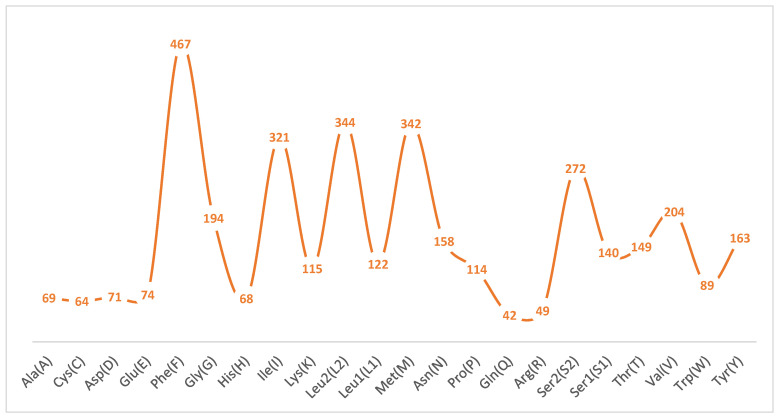
Amino acid composition analysis of protein-coding genes.

The A+T content at the first, second, and third codon positions is 72.3%, 68.7%, and 87.7%, respectively, reflecting a strong preference for A or U at synonymous sites. Relative synonymous codon usage (RSCU; Figure 2) further highlights this bias: codons ending in A or U are markedly overrepresented, with UUU (Phe) exhibiting the highest RSCU value, followed by AUU (Ile) and AUA (Met). Correspondingly, amino acid frequency analysis (Figure 3) shows that Phe is the most abundant residue (12.86%), followed by Leu2 (9.47%) and Met (9.42%). In contrast, Gln, Arg, Cys, His, Ala, and Asp each account for less than 2% of residues, their cognate codons typically terminating in C or G, further corroborating the genome-wide A+T bias.

### 3.3. Transfer and Ribosomal RNA Genes

**Figure 4:**
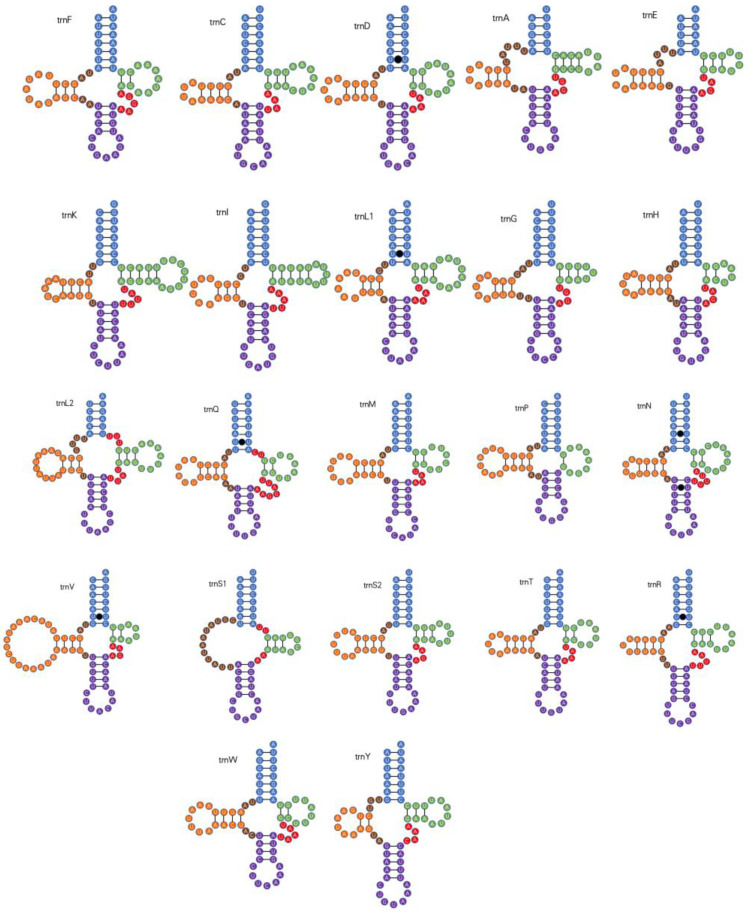
Secondary structure models for the 22 mitochondrial tRNAs encoded by the *P. arcuata*. mitogenome.

*P. arcuata* harbors 22 canonical mitochondrial tRNA genes ranging from 56 bp (*trnS1*) to 73 bp (*trnV*) in length (Table 2); their combined A+T content is 79% (Table 3). With the exception of *trnS1*, all tRNAs fold into the standard clover-leaf secondary structure, in which the dihydrouridine (DHU) arm is reduced to a simple loop. Across the 22 tRNAs, seven non-canonical base pairs were identified, all of the UU type (Figure 4).

The *rrnL* (1180 bp) is situated between *trnL1* and *trnV*, whereas the *rrnS* (734 bp) resides between *trnV* and the control region; this arrangement is congruent with that reported for other Cicadellidae species. The two rRNA genes exhibit an average A+T content of 80.8%, mirroring the values reported by Wang et al. [24] for two congeners and making them, aside from the control region, the most A+T-rich coding segments of the mitogenome.

### 3.4. Control Region

The control region of *P. arcuata* was found to be 250 bp in length, positioned between *rrnS* and *trnI*, and exhibits a pronounced A+T content of 88% (Table 2).

### 3.5. Phylogenetic Relationships

**Figure 5:**
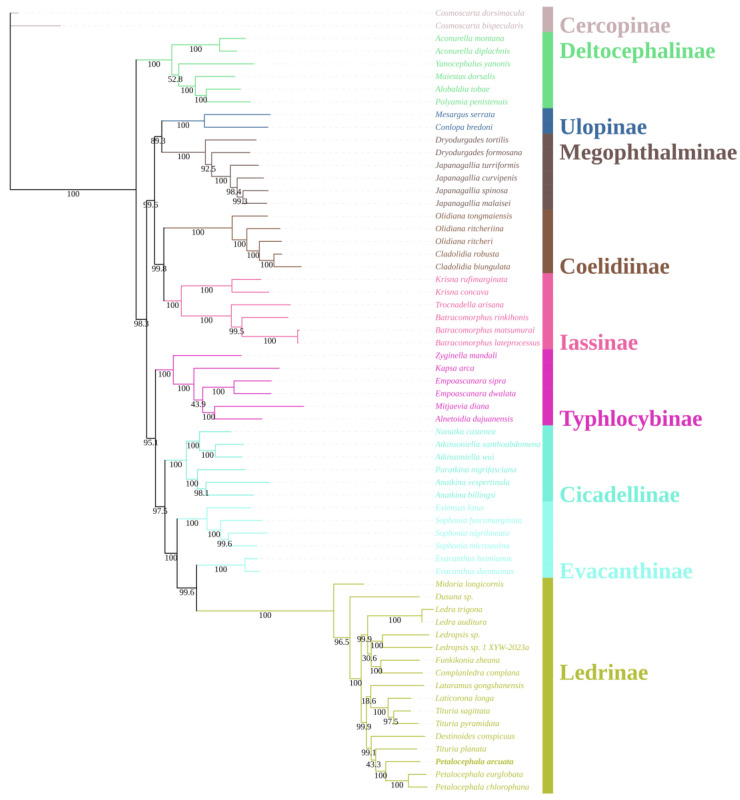
Phylogenetic tree Cicadellidae species inferred via maximum likelihood analyses of the whole mitogenomes sequences.

**Figure 6:**
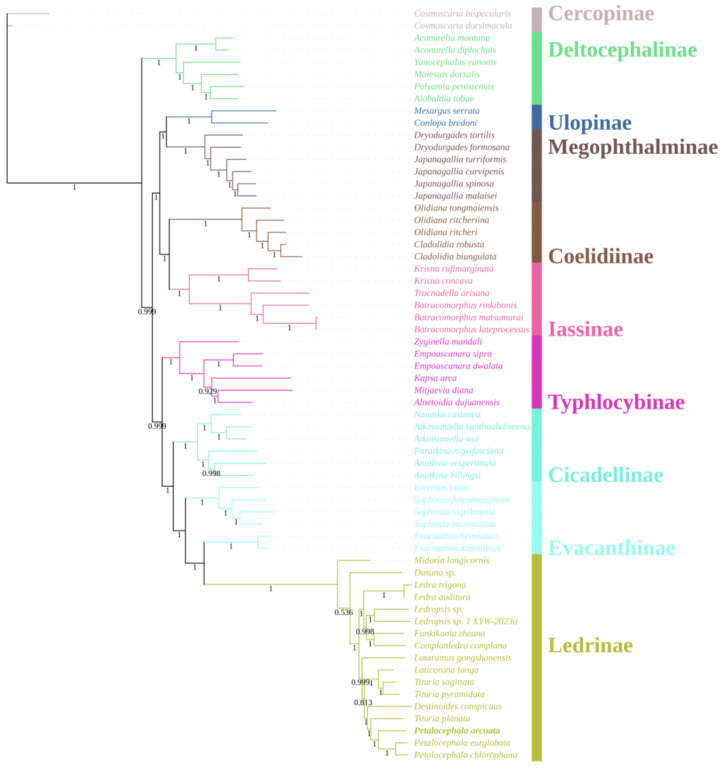
Phylogenetic tree of Cicadellidae species inferred via Bayesian analyses of the whole mitogenomes sequences.

A concatenated nucleotide matrix comprising the 13 mitochondrial protein-coding genes and both ribosomal RNA genes was assembled for 60 ingroup taxa representing nine subfamilies of Cicadellidae, together with two Cercopoidea species (*C. dorsimacula* and *C. bispecularis*) as outgroups. Phylogenetic reconstruction was conducted under maximum-likelihood (ML) (Figure 5) and Bayesian-inference (BI) (Figure 6) frameworks. The two optimal trees were topologically congruent and exhibited uniformly high nodal support (ML bootstrap, BS; Bayesian posterior probability, PP). At the family level, eight of the nine sampled subfamilies were recovered as monophyletic with strong support; the sole exception was Evacanthinae. The backbone topology can be summarized as (((((Ledrinae + Evacanthinae) Evacanthinae) + Cicadellinae) + Typhlocybinae) + ((Iassinae + Coelidiinae) + (Megophthalminae + Ulopinae))) + Deltocephalinae, where Evacanthinae is resolved as the well-supported sister group of Ledrinae (BS = 100; PP = 1.00). Evacanthinae itself forms two deeply divergent lineages (BS = 99.6; PP = 1.00): lineage I joins Ledrinae, Cicadellinae and Typhlocybinae, whereas lineage II is allied to Iassinae, Coelidiinae, Megophthalminae and Ulopinae. Deltocephalinae is placed as the basal clade of the family, consistent with earlier studies [10].

Within Ledrinae, the ML and BI topologies are virtually identical: *Ledra* and *Ledropsis* each constitute highly supported monophyletic clades. Two additional, strongly supported sister-group relationships were identified: (i) *Laticorona* is recovered as the sister lineage to *Tituria* (BS = 100; PP = 1.00), and (ii) *Destinoides* forms the sister lineage to the composite clade (*Petalocephala* + *Tituria*) (BS = 100; PP = 1.00). These results not only reinforce the monophyly of *Petalocephala* but also concur with the revision by Wang et al. [24], which removed *L. gongshanensis* from *Petalocephala* and erected a separate genus for the species.

## 4. Conclusions

In this study, we sequenced and characterized the complete mitochondrial genome of *P. arcuata*, a representative species of the Ledrinae. The circular mitogenome (14,491 bp) contains the typical 37 mitochondrial genes and an A+T-rich control region. Comparative analysis revealed that all protein-coding genes (PCGs) employ standard ATN initiation codons, with the notable exception of *ND5* which utilizes TTG as its start codon. Secondary structure predictions showed that 21 of the 22 tRNAs adopt the canonical cloverleaf configuration, while tRNA-Ser1 (AGN) exhibits structural simplification through the loss of its DHU arm.

To clarify intrafamilial relationships within Cicadellidae, we compiled a concatenated dataset of the nucleotide sequences of 13 mitochondrial protein-coding genes (PCGs) and two rRNA genes from 62 representative species spanning nine subfamilies. Phylogenetic trees were reconstructed under both a Bayesian framework (MrBayes) and a maximum-likelihood framework. The resulting topologies were highly congruent, with most nodes exhibiting strong statistical support. Importantly, our analyses unequivocally corroborate the monophyly of the genus *Petalocephala*.

## Figures and Tables

**Table 1:** Sequences used in this study.

Subfamily	Species	GenBank Accessionnumber	Length (bp)
Cercopinae	*Cosmoscarta dorsimacula*	NC_040115	15,677
	*Cosmoscarta bispecularis*	KP064511	15,426
Cicadellinae	*Anatkina billingsi*	OR840943	16,967
	*Anatkina vespertinula*	ON597623	15,559
	*Atkinsoniella wui*	NC_062852	15,159
	*Atkinsoniella xanthoabdomena*	NC_062853	15,463
	*Nanatka castenea*	ON584003	14,953
	*Paratkina nigrifasciana*	NC_069999	16,194
Coelidiinae	*Cladolidia biungulata*	NC_067788	15,247
	*Cladolidia robusta*	NC_067789	15,376
	*Olidiana ritcheri*	NC_057965	15,372
	*Olidiana ritcheriina*	MN780581	15,205
	*Olidiana tongmaiensis*	NC_057966	15,363
Deltocephalinae	*Aconurella diplachnis*	OK105069	15,598
	*Aconurella montana*	OK105070	15,637
	*Alobaldia tobae*	KY039116	16,026
	*Maiestas dorsalis*	NC_036296	15,352
	*Polyamia penistenuis*	OP972729	15,416
	*Yanocephalus yanonis*	NC_036131	15,623
Evacanthinae	*Evacanthus heimianus*	MG813486	15,806
	*Evacanthus danmainus*	MN227166	15,343
	*Extensus latus*	OQ957162	16,032
	*Sophonia fuscomarginata*	OR727345	15,796
	*Sophonia microstaina*	OR727343	15,925
	*Sophonia nigrilineata*	OR727342	15,610
Iassinae	*Batracomorphus lateprocessus*	NC_045858	15,356
	*Batracomorphus matsumurai*	NC_080325	15,009
	*Batracomorphus rinkihonis*	NC_080328	15,385
	*Krisna concava*	NC_046067	14,304
	*Krisna rufimarginata*	NC_046068	14,724
	*Trocnadella arisana*	NC_036480	15,131
Ledrinae	*Complanledra complana*	MZ333274	15,031
	*Destinoides conspicuus*	MZ333275	14,913
	*Dusuna* sp.	MZ333276	15,198
	*Funkikonia zheana*	MZ333278	15,062
	*L. gongshanensis*	MW018818	14,969
	*Laticorona longa*	MZ333279	15,540
	*Ledra auditura*	MK387845	16,094
	*Ledra trigona*	MG813491	16,094
	*Ledropsis sp.*	MZ333280	14,916
	*Ledropsis sp. 1*	MZ853174	15,019
	*Midoria longicornis*	MZ333281	15,130
	*P. arcuata*	PP893239	14,491
	*Petalocephala chlorophana*	NC_051527	14,927
	*P. eurglobata*	MW018817	14,834
	*Tituria planata*	MZ333277	15,088
	*Tituria pyramidata*	NC_046701	15,331
	*Tituria sagittata*	NC_051528	14,918
Megophthalminae	*Dryodurgades formosana*	NC072571	15,587
	*Dryodurgades tortilis*	NC_086634	15,674
	*Japanagallia curvipenis*	NC_082332	15,356
	*Japanagallia malaisei*	OQ658733	15,575
	*Japanagallia spinosa*	NC_035685	15,655
	*Japanagallia turriformis*	NC_082334	15,717
Typhlocybinae	*Alnetoidia dujuanensis*	NC_069628	15,375
	*Empoascanara dwalata*	MT350235	15,271
	*Empoascanara sipra*	NC_048516	14,827
	*Kapsa arca*	NC_069862	15,594
	*Mitjaevia diana*	OK448489	16,589
	*Zyginella mandali*	ON055365	15,166
Ulopinae	*Mesargus serrata*	MG813495	15,399
	*Conlopa bredoni*	OR187397	14,731

**Table 2:** Mitogenomic organization of *P. arcuata*.

Gene	Location		Size (bp)	IN	Codon		Strand
	From	To			Start	Stop	
*trnI*	1	67	67				H
*trnQ*	65	131	67	−3			L
*trnM*	132	195	64				H
*ND2*	196	1170	975		ATT	TAA	H
*trnW*	1171	1234	64				H
*trnC*	1227	1290	64	−8			L
*trnY*	1293	1355	63	2			L
*COI*	1360	2893	1534	4	ATG	T	H
*trnL2*	2894	2961	68				H
*COII*	2962	3640	679		ATT	T	H
*trnK*	3641	3711	71				H
*trnD*	3715	3778	64	3			H
*ATP8*	3779	3931	153		ATT	TAA	H
*ATP6*	3931	4581	651	−1	ATA	TAA	H
*COIII*	4583	5362	780	1	ATG	TAA	H
*trnG*	5366	5426	61	3			H
*ND3*	5427	5780	354		ATT	TAA	H
*trnA*	5784	5844	61	3			H
*trnR*	5845	5907	63				H
*trnN*	5908	5971	64				H
*trnS1*	5969	6024	56	−3			H
*trnE*	6032	6092	61	7			H
*trnF*	6094	6156	63	1			L
*ND5*	6157	7819	1663		TTG	T	L
*trnH*	7820	7879	60				L
*ND4*	7879	9168	1290	−1	ATG	TAA	L
*ND4L*	9162	9437	276	−7	ATA	TAA	L
*trnT*	9443	9504	62	5			H
*trnP*	9505	9564	60				L
*ND6*	9573	10,064	492	8	ATT	TAA	H
*Cytb*	10,057	11,193	1137	−8	ATG	TAG	H
*trnS2*	11,192	11,256	65	−2			H
*ND1*	11,247	12,188	942	−10	ATA	TAA	L
*trnL1*	12,189	12,254	66				L
*rrnL*	12,255	13,434	1180				L
*trnV*	13,435	13,507	73				L
*rrnS*	13,508	14,241	734				L
*CR*	14,242	14,491	250				H

**Table 3:** Base composition of the mitochondrial genome of *P. arcuata*.

Regions	Size (bp)	T (U)	C	A	G	AT (%)	GC (%)	AT Skew	GC Skew
Full genome	14,491	48.9	9.8	28.5	12.9	77.4	22.7	−0.264	0.136
PCGs	10,923	46.5	11	29.8	12.8	76.3	23.8	−0.219	0.073
tRNAs	1407	41.3	8.6	37.7	12.4	79	21	−0.045	0.18
rRNAs	1914	32.7	8.3	48.1	10.9	80.8	19.2	0.19	0.136
1st codon position	3641	38.7	10.8	33.6	16.8	72.3	27.6	−0.07	0.215
2nd codon position	3641	49.4	16.6	19.3	14.7	68.7	31.3	−0.439	−0.06
3rd codon position	3641	51.3	5.6	36.4	6.7	87.7	12.3	−0.17	0.091
Control region	250	50.8	4	37.2	8	88	12	−0.155	0.333

## Data Availability

The mitogenome of *Petalocephala arcuata* has been deposited in the GenBank database under the accession number PP893239. The genomic Illumina sequencing data were deposited in the NCBI Sequence Read Archive (SRA) database under accession numberPP893239.
